# Protocol for the rapid intravenous *in ovo* injection of developing amniote embryos

**DOI:** 10.1016/j.xpro.2023.102324

**Published:** 2023-05-19

**Authors:** Rory L. Cooper, Gabriel Santos-Durán, Michel C. Milinkovitch

**Affiliations:** 1 Laboratory of Artificial & Natural Evolution (LANE), Department of Genetics and Evolution, University of Geneva, 1211 Geneva, Switzerland; 2 SIB Swiss Institute of Bioinformatics, Geneva, Switzerland

**Keywords:** Developmental Biology, Model Organisms, Evolutionary Biology

## Abstract

We present a technique for precise drug delivery into the vascular system of developing amniote embryos via injection into chorioallantoic veins underlying the eggshell membrane. We describe steps for incubating and candling eggs, removing the shell to expose underlying veins, and precise intravenous injection. In addition to chicken embryos, this protocol is applicable to other amniote species that lay hard-shell eggs, including crocodiles and tortoises. This technique is rapid, is reproducible, is of low cost, and will provide an important resource for developmental biologists.

For complete details on the use and execution of this protocol, please refer to Cooper & Milinkovitch.^1^

## Highlights

• Technique for the intravenous injection of amniote embryos within hard-shelled eggs
• Description of experimental manipulation of chicken, crocodile, and tortoise eggs
• Protocol is precise, fast, and of low cost, making it widely accessible to researchers
• Broadly applicable to studies in developmental biology and beyond

## Abstract (editor highlights)

Publisher’s note: Undertaking any experimental protocol requires adherence to local institutional guidelines for laboratory safety and ethics.

## Graphical abstract

**Figure:**
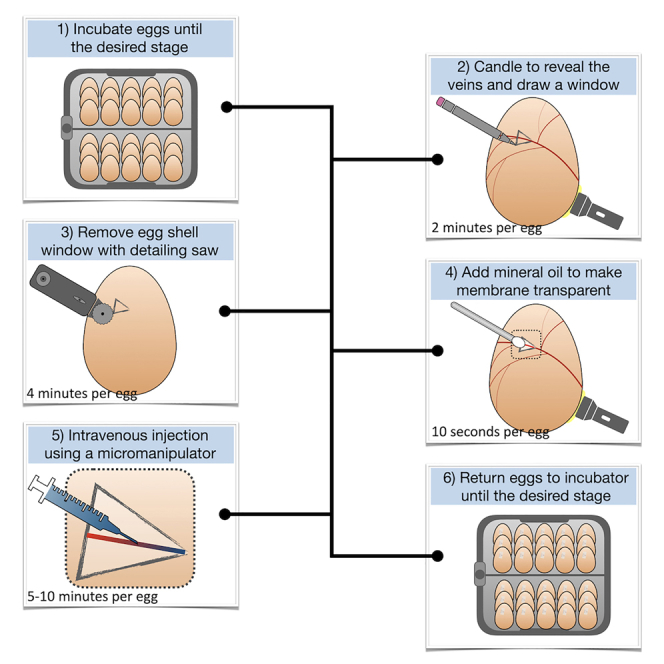

## Before you begin

### Institutional permissions

**Timing: 10 days prior to injections (for step 1)**

**Timing: Any time prior to intravenous injections (for step 2)**

**Timing: Immediately prior to injections (for step 3)**

Maintenance of and experiments with all chicken embryos were approved by the Geneva Canton ethical regulation authority (authorization GE10619B) and performed according to Swiss law. Readers must acquire all necessary training and permissions relevant to their institution prior to undertaking any animal experimentation.

1. Incubation of fertilized chicken embryos.
  a. Incubate eggs to achieve the desired stage on the day of injection. This protocol works well in the chicken from embryonic day 10 (E10) onwards. In tortoise and crocodile species, this protocol works well from E40 onwards.
  b. The upwards facing side of the egg should be marked with a pencil to ensure that its original orientation is maintained when returning eggs to the incubator.
  c. Incubation temperature should be maintained at 37.5°C, with a relative humidity of approximately 40%–50%, achieved by placing a tray of water in the incubator.
  d. Eggs should be turned every 2 h, as this is important for embryonic vascular development.

***Note:*** Most chicken egg incubators feature an automatic turning function. Incubation conditions for other amniote species vary.

**CRITICAL:** Crocodile and tortoise eggs should not be rotated and should be incubated at 31°C in moist vermiculite.

2. Preparation of paraffin egg mounts (Figure S1).
  a. Melt paraffin wax (e.g., Paraplast) in an oven at approximately 60°C.
  b. Pour the melted wax into a petri dish. A dish of any size may be used, providing that it is larger than the egg.
  c. When the wax begins to harden, make an impression of an egg of the desired size.
  d. Allow the paraffin to set completely. This will be used as a mount to hold the eggs during windowing and injection. These mounts can be reused indefinitely.
3. Preparation of drug solutions
  a. Prepare a 2.5% Patent blue solution in ddH_2_0.
  b. Solutions should be prepared and stored according to manufacturers’ instructions.
    ***Note:*** For example, we have previously treated chicken embryos at E11 with 30 μL aliquots of dimethyl sulfoxide (DMSO) containing 200 μg Smoothened agonist (SAG). Control solutions should be prepared, for example 30 μL aliquots of DMSO.
  c. Patent blue dye should now be added to enable visualization of the solution during injection, for example 0.5 μL of a 2.5% Patent blue solution (dissolved in ddH_2_0).
    **CRITICAL:** To avoid degradation of drugs, solutions should be prepared immediately prior to undertaking injections, and time kept at room temperature should be minimized.

## Key resources table

**Table:** 

REAGENT or RESOURCE	SOURCE	IDENTIFIER
**Experimental models: Organisms/strains**		

Fertilized chicken (*Gallus gallus*) eggs	La Prairie Chicken Farm	La Prairie 9, 1721 Cournillens, Switzerland
Fertilized crocodile (*Crocodylus niloticus*) eggs	Seronera Crocodile Farm	Hazyview, 1242, South Africa
Fertilized tortoise (*Centrochelys sulcata*) eggs	Private breeders	Various

**Other**		

Automated chicken egg incubator (choose model with automatic egg turning function)	Various (such as Brinsea Ova Easy Advance)	MJ1023C
Candling torch	Various (for example Power Lux LED eggtester)	Various
Stereo zoom dissecting microscope	Various (for example Kern Optics OZL-44)	Various
Micromot 50/E mill/drill unit	Proxxon, sold at www.bauhaus.ch (60086572)	60086572
Cutting disks for Micromot 50/E (0.7mm thickness) – included with the above drill unit	Proxxon, sold at www.bauhaus.ch	60086572
MM33 Micromanipulator	Marzhauser, sold at www.marzhauser.com	00-42-103-0000
Hamilton syringe with luer tip, 50 μL (for crocodiles/tortoises)	Sigma-Aldrich	20701
TSK STERiJECT hypodermic needle 33G *×* 13mm (luer connection) or similar (for crocodiles/tortoises)	Dermat, sold at www.dermat.be	PRE-33013
Hamilton syringe, 700 series, with removable needles (100 μL) (for chicken)	SigmaAldrich	20790-U
Hamilton 32G Small Hub RN Needle custom length (2 inches) (for chicken)	Hamilton (www.hamiltoncompany.com)	7803-05
Disposable insulin syringes – BD Micro-Fine Plus, 0.3 mL, 30G *×* 8 mm, or similar (for EdU injections – optional)	Apo24, sold at www.apo24.ch	320837
Petri dish 92 × 16 mm	Sarstedt	82.1473.001
Paraplast paraffin wax, 1 kg	Sigma-Aldrich	327204-1KG
Cotton buds for applying mineral oil	Various	Various
Clear adhesive tape	Various	Various
Fine forceps	Various	Various
Vermiculite for reptilian egg incubation	Various (for example www.amazon.co.uk)	Various

**Chemicals, peptides, and recombinant proteins**		

Mineral oil, 500 mL	Sigma-Aldrich	M5904-500ML
Dimethyl sulfoxide (DMSO), 100 mL	Sigma-Aldrich	D8418-100ML
Smoothened Agonist (SAG), 5 mg. This is provided as an example treatment, but any chemicals, peptides or recombinant proteins can be injected in suitable solvents.	Selleckchem	S6384
Patent Blue VF, 25 g, dissolved in a 2.5% solution in ddH_2_0	Sigma-Aldrich	198218-25G
Ethanol (EtOH), 500 mL, used for 70% EtOH cleaning solution	Sigma-Aldrich	51976-500ML-F
EdU-Click kit for labeling and detection of proliferating cells (optional)	Sigma-Aldrich	BCK-EDU555
Phosphate buffered saline (PBS)	Sigma-Aldrich	P4417-50TAB
Paraformaldehyde (PFA), 1KG	Sigma-Aldrich	16005-1KG-R

## Step-by-step method details

### Step 1: Candle eggs to identify a suitable chorioallantoic vein for injection

**Timing: Approximately 2 min per egg**

**Figure 1:**
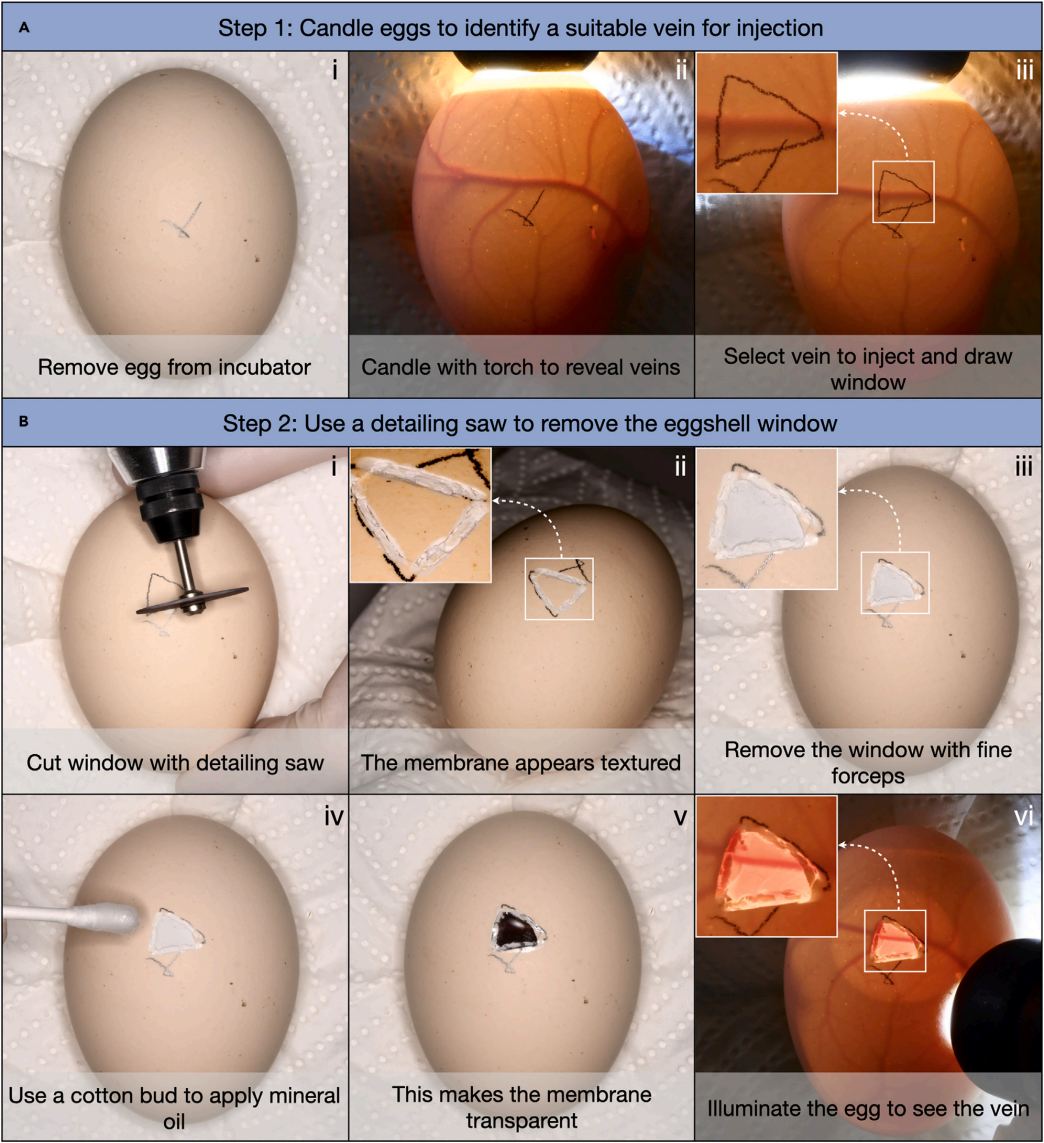
Preparing eggs for intravenous injection into the chorioallantoic veins (A) Eggs are candled to identify a suitable chorioallantoic vein to inject (Ai-ii), and a small window is drawn around this vein (Aiii). (B) This window is then removed using a detailing saw, whilst leaving intact the membrane underneath the egg (Bi-iii). After the application of mineral oil, the vein becomes clearly visible when illuminated with a candling torch (Biv-vi). Images presented here show the preparation of a chicken egg for intravenous injection.

Once eggs have reached the desired developmental stage, they should be removed from the incubator and candled to identify a suitable chorioallantoic vein for injection (Figure 1A). Larger veins are easier to inject. This step should be undertaken in the dark to enable clear visualization of veins with a candling torch. For reptilian species that we have tested (including the Nile crocodile and various tortoise species), veins are typically visible from approximately halfway through the total incubation period. We have previously injected chicken embryos between E10 and E14, and we have injected tortoise and crocodile embryos between E40 and E60. See ‘Methods video S1: Protocol for the rapid intravenous in-ovo injection of developing amniote embryos’ for a clear demonstration of the entire protocol.

1. Remove eggs from the incubator, ensuring that the upward facing side remains in the same orientation (Figure 1Ai).
2. Place the egg in the paraffin egg mount covered with damp tissue paper.
3. Clean the upper surface of the egg by wiping gently with a 70% EtOH solution.
4. Use a candling torch to illuminate the egg, thereby visualizing the chorioallantoic veins (Figure 1Aii).

***Note:*** Moving the torch around the egg will illuminate different veins, allowing the largest veins to be selected.

5. Once an appropriate vein has been selected, draw a triangular window around the vein using a pencil, approximately 6 × 6 × 6 mm in size (Figure 1Aiii).
6. Repeat this process for all eggs, and then return them to the incubator until you are ready to proceed to the next stage.

Methods video S1. Protocol for the rapid intravenous in-ovo injection of developing amniote embryos Video demonstrating all steps required for the successful execution of this protocol.

### Step 2: Use a detailing saw to remove an eggshell window and apply mineral oil

**Timing: Approximately 4 min per egg**

Using the detailing saw, carefully cut through the eggshell without damaging the underlying membrane (Figure 1B). Directing light from the candling torch perpendicular to the surface of the egg should help to visualise when the membrane has been reached, as there is a sudden change in material texture once the membrane has been reached, with a visible increase in material roughness once the eggshell has been fully penetrated.

7. Remove an egg from the incubator and use the detailing saw to carefully cut through the window that was drawn around a selected vein in the previous step (Figure 1Bi).

***Note:*** When the membrane has been reached, its surface will appear whiter and rougher in texture than the eggshell (Figure 1Bii).

**CRITICAL:** Ensure that the egg membrane is not damaged. It is advisable to incubate more eggs than required to allow for wastage during this step.

***Note:*** Piercing of the membrane can enable air bubbles in enter the egg, which may dislodge the chorioallantoic veins.

8. Carefully remove the eggshell window with fine forceps, thereby exposing the underlying membrane (Figure 1Biii).
9. Repeat this process for all eggs.
10. Using a cotton bud, gently apply a small amount of mineral oil to the exposed membrane of the windowed eggs.

***Note:*** This will increase the transparency of the membrane, enabling clear visualization of the vein (Figures 1Biv to 1Bvi). For reptilian eggs (such as crocodiles and tortoises) with thicker membranes, more mineral oil and persistent rubbing of the membrane will be required to achieve transparency. We have not observed adverse effects of mineral oil upon embryonic development.

### Step 3: Intravenous injection into the selected chorioallantoic vein

**Timing: Approximately 5–10 min per egg**

**Figure 2:**
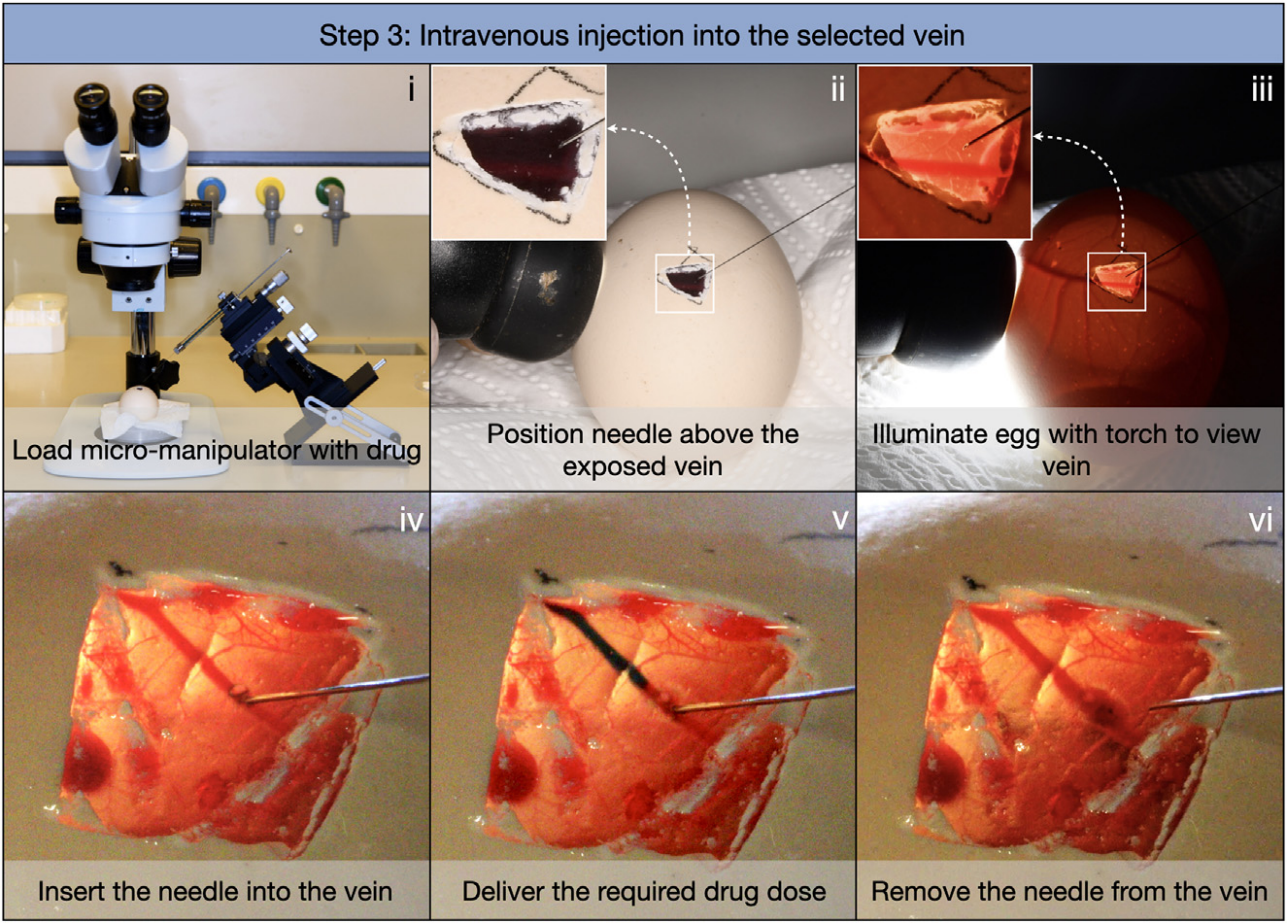
Intravenous injection of windowed eggs Place the windowed egg on the paraffin egg mount underneath a dissecting microscope and load the micro-manipulator with the drug (i). Use the micro-manipulator to position the needle directly above the chorioallantoic vein (ii). By illuminating the egg with a candling torch, the vein will be visible (iii). Use the micro-manipulator to carefully penetrate the vein (iv) and deliver the required drug dose (v), before removing the needle from the vein (vi). See also Methods video S1. Images presented here show the intravenous injection of a chicken egg.

Windowed eggs can now be injected with a chosen drug, directly into the chorioallantoic vein underlying the egg membrane (Figure 2). For injections in chicken eggs, a long and flexible Hamilton needle (e.g., 32G) is suitable as the membrane is very thin. However, for species with thicker membranes (such as tortoises and crocodiles), shorter and less flexible hypodermic needles can be used (see key resources table). This step should be undertaken in the dark to enable clear visualization of the veins with the candling torch.

11. Place the egg in the paraffin egg mount on the stage of a dissecting microscope (Figure 2i).

***Note:*** The addition of damp tissue paper to the egg mount helps to increase egg stability.

12. Hold the candling torch against the side of the egg, in the position that most clearly illuminates the chorioallantoic vein selected for injection, when viewed through the dissecting microscope (Figures 2ii and 2iii).

***Note:*** If required, re-apply mineral oil to the membrane to increase transparency.

13. Load the syringe with your chosen drug solution, ensuring that no air bubbles are present, as bubbles may cause blockage of blood vessels and subsequent embryonic mortality.

***Note:*** For chicken embryos from E10 onwards, 30 μL of solution can be injected whilst avoiding adverse effects. For crocodile and tortoise eggs, 30–50 μL of solution can be injected. Use the micromanipulator to position the needle directly above the vein, so that it will enter at an angle of approximately 45° (Figures 2ii and 2iii).

**CRITICAL:** Use the micromanipulator to move the needle forwards and penetrate the vein (Figure 2iv), ensuring that the needle does not exit the lower surface of the vein. Use the syringe to deliver the required dose of your chosen drug; the patent blue dye will allow clear visualization of the solution entering the vein (Figure 2v and Methods video S1).

14. Use the micromanipulator to gently retract the needle (Figure 2vi).

***Note:*** For large veins which are typically more elastic, bleeding from the site of injection is generally minimal.

15. Clean around the window by wiping with 70% EtOH to remove excess mineral oil.
16. Use clear adhesive tape to cover the window and exposed membrane, thereby preventing infection. Ensure that the eggs are clearly labeled with a pencil and return to the incubator.

***Note:*** If chicken embryos are incubated until the hatching stage, it is important to turn off the incubators rocking function from E18 onwards and maintain humidity throughout.

### Step 4: Embryo dissection and fixation

**Timing: Approximately 5 to 10 min per embryo**

Embryos should be fixed at the desired developmental stages. For example, to investigate the emergence of ectopic feather buds resulting from SAG treatment at E11 ^1^, embryos were fixed at either E16 or E20, or allowed to hatch. If required, embryos can also be injected with EdU to label proliferating cells prior to fixation. Similarly, we have injected and fixed both crocodiles and tortoises between E40 and E60 to investigate scale formation.

17. Remove eggs from the incubator and use fine forceps to remove the adhesive tape.
18. If required, EdU solution can be injected directly into the chicken embryo through the original window, using a disposable insulin syringe. If access to the embryo through the original window is insufficient, the window can be enlarged using fine scissors. Injection of 150 μL of a 10 mg/mL EdU solution, followed by incubation for 2 h prior to fixation, is sufficient to label proliferating cells in chicken embryos between E16 and E20. Injection of 200 μL of a 10 mg/mL EdU solution followed by 3 h incubation is sufficient to label proliferating cells in crocodile and tortoise embryos from E45 until hatching.
19. Use small scissors to remove the top of the egg, starting from the window. The embryo can then be carefully removed using a blunt instrument such as a metal spoon, and placed into a dish of PBS (at room temperature). Embryos should immediately be decapitated following removal from the egg.

***Note:*** Hatched animals should be euthanized in accordance with local legislation, for example with injection of Sodium pentobarbital.

20. Dissect the tissue of interest and rinse in fresh PBS before placing the sample into a fixative, if required. After fixation, continue with subsequent analysis as required.

***Note:*** Overnight fixation in 4% paraformaldehyde in PBS, followed by graded dehydration into ethanol is sufficient for the fixation of chicken, tortoise and crocodiles. Additionally, fixation and long-term storage in 10% neutral buffered formalin can be used to avoid subsequent dehydration of samples, which may result in some morphological deformation of embryonic samples.

## Expected outcomes

This protocol was first described in the American Journal of Pathology over 80 years ago,^2^ prior to some small modifications, such as the inclusion of a micromanipulator, in the subsequent years.^3^^,^^4^ Despite being a powerful tool for manipulating amniote embryonic development, intravenous *in-ovo* injection of developing embryos remains under-utilised in recent research. Here, we provide a clear step-by-step description of this method, thereby making this historical technique easily accessible for modern day researchers. Furthermore, we provide specific adaptations to facilitate experimentation in non-model amniotes such as tortoises and crocodiles, thereby broadening the applicability of this method to encompass a greater diversity of species and developmental systems. This protocol has abundant potential outcomes and applications. It is low-cost, making it highly accessible to scientists working in diverse research environments. Furthermore, it is both precise and fast, meaning that it can be employed for undertaking relatively large treatment trials. Indeed, with practice, it is possible to window and inject over 40 chicken eggs per h.

This protocol has broad implications for researchers studying the embryonic development of non-model amniote species laying hard-shelled eggs. Clutches of stage-specific fertilized reptilian eggs are typically less widely available than fertilized chicken eggs, in part due to seasonal breeding habits. Although some species-specific biological responses are to be expected, we have found that results from trialing different drugs and specific drug dosages in chicken embryos are frequently transferable to reptilian embryos, when adjusted to account for differences in embryonic weight. Therefore, experiments can be refined using relatively abundant and inexpensive chicken embryos, prior to the treatment of less abundant reptilian embryos. This can dramatically increase research efficiency when embryonic sample numbers are limited.

Multiple previous studies have treated developing chicken embryos with injections of both drugs and replication competent avian sarcoma virus (RCAS), delivered directly into the amniotic cavity.^5^^,^^6^^,^^7^^,^^8^^,^^9^^,^^10^ However, because such an approach relies upon passive diffusion of drugs into the embryo, results can exhibit substantial variation, depending on both the precise location of drug delivery within the amniotic cavity, and the relative position of the embryo. Our method of intravenous drug delivery into the chorioallantoic veins underlying the eggshell membrane is much more precise, potentially leading to more robust experimental results than are achievable by other non-intravenous routes of inoculation. Therefore, this technique has the potential to dramatically increase the standardization of *in vivo* experimentation in the chicken embryo.^11^

We have used this technique to study the evolution and development of integumentary appendages in the chicken,^1^ as well as crocodile and tortoise embryos (in preparation), by using a combination of small molecules and growth factors to target gene signalling,^1^ cell proliferation and tissue mechanics. Specifically, we have shown that agonism of the sonic hedgehog (Shh) signalling pathway *via* intravenous delivery of SAG is sufficient to induce a permanent transition of skin appendage fate (from scales to feathers) in regions of the chicken foot and shank.^1^ Although this technique of intravenous injection has previously been used within biomedical and immunological research,^12^^,^^13^ it has not been employed within the field of developmental biology despite its broad relevance and applicability.

## Limitations

There are various limitations associated with this protocol. First, although a wide range of amniote eggs can be used, they must exhibit a hard shell that can be drilled and removed from the underlying membrane. This is not the case for most lizard and snake species which typically exhibit soft-shelled eggs. Furthermore, the vascular system underlying the egg surface must be well developed, meaning that the chorioallantoic veins are sufficiently large and elastic enough to inject without subjecting them to significant damage and bleeding. Experimenting with different needle sizes may help to overcome such problems (see ‘troubleshooting’ for further information). Additionally, the chorioallantoic veins must be well adhered to the membrane underlying the eggshell, otherwise the lack of tension can make them very difficult (although still feasible) to inject. With chicken eggs incubated at our research facility, this was not a problem. However, a small proportion of developing tortoise and crocodile eggs transported to our laboratory from external facilities exhibited detached veins that were very difficult to inject. Finally, some amniotes (for example tortoises) exhibit diverse rates of development, even when originating from the same clutch of eggs and incubated under the same conditions. This can increase variability in experimental results and should therefore be considered when designing experiments.

## Troubleshooting

### Problem 1: Piercing the inner eggshell membrane (during step 2)

A common problem that can arise whilst implementing this protocol is piercing of the inner eggshell membrane whilst drilling the window or removing the freshly cut window with fine forceps.

### Potential solution

Providing that air bubbles have not entered the incision, it is often possible to use a small amount of a strong adhesive glue (for example UHU Superglue) to seal the hole in the membrane, before locating a new chorioallantoic vein and making a new window. However, if air bubbles do enter the incision, they will diffuse to the highest point of the allantoic cavity and can physically dislodge the chorioallantoic veins whilst they travel. This detachment of the veins from the membrane makes subsequent injection very challenging. If injection is not possible, the egg should be discarded, unless the embryo can be fixed and used for alternative studies.

### Problem 2: Selecting a suitable chorioallantoic vein

Sometimes, veins that appeared to be suitable for injection during candling are too small to inject after the window has been made. This is often the case for eggs with thick shells, such as crocodile eggs, in which veins are less clearly visible during candling.

### Potential solution

If this is the case, it is possible to locate a new vein and create a new window. This is often preferable to attempting the injection of a very small vein, which can result in substantial damage and bleeding. We have previously windowed individual eggs up to four times without observing any notable adverse developmental effects.

### Problem 3: Selection of an appropriate needle

The stiffness of the inner eggshell membrane varies substantially across species.

### Potential solution

In chickens, the membrane is very thin, meaning that long, thin, and flexible needles can be used to deliver drugs directly into the chorioallantoic veins, such as 32G needles of 2 inches in length. However, in crocodiles the membrane is substantially thicker, meaning that shorter and stiffer needles are required, such as 30G needles of 8 mm in length. Experimenting with different needle types is an important stage of optimizing this protocol for the eggs of a specific species. See the ‘key resources table’ for specific advice regarding needle types.

### Problem 4: Transparency of the eggshell membrane

The transparency of the eggshell membrane varies among different species.

### Potential solution

For chicken eggs, the application of mineral oil should immediately make the membrane transparent. However, for other species with thicker and less transparent membranes, the application of more abundant mineral oil and extensive rubbing with the cotton bud is often required.

### Problem 5: Bleeding from the chorioallantoic veins

Both the thickness and elasticity of chorioallantoic veins can vary substantially both among eggs of different developmental stages and among eggs from different species. This can be problematic, as injecting thin and inelastic veins can result in substantial bleeding and, subsequently, embryo mortality.

### Potential solution

We have noted that increasing incubation humidity may increase vein dilation in certain species (for example in the crocodile). If small vein size is problematic, experimenting with different needle types may also provide a solution. Furthermore, alternative drug delivery devices can be considered, for example, thin glass capillary needles.

## Resource availability

### Lead contact

Further requests for information should be directed to the lead contact, Michel Milinkovitch (Michel.Milinkovitch@unige.ch).

### Materials availability

This study did not result in the generation of new, unique reagents.

### Data and code availability

This study did not result in the generation of datasets or codes.
