# Supplementary material for: Protocol for the rapid intravenous in ovo injection of developing amniote embryos
Source: STAR Protoc. 2023 May 19;4(2):102324. doi: 10.1016/j.xpro.2023.102324 (PMC10209871; doi:10.1016/j.xpro.2023.102324)
Supplement: Document S1. Figures S1 [file mmc1.pdf]

Supplemental Figures

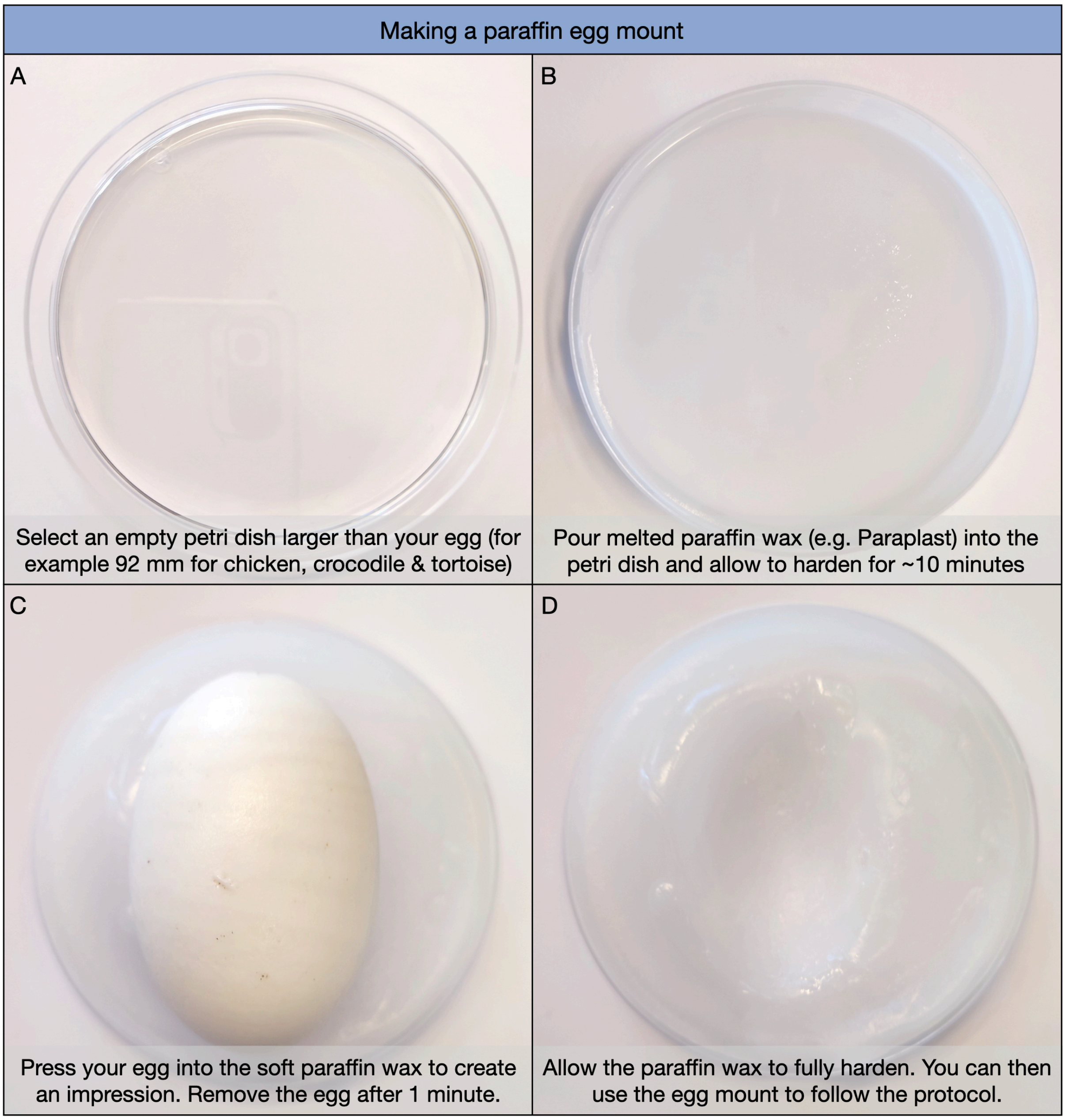

Supplemental figure S1: Making a paraffin egg mount (Step: 'Before you begin'). (A) To create an egg mount to stabilise the egg, first select an empty petri dish larger than the egg that you will be injecting. (B) Fill this petri dish with paraffin wax to approximately 2/3rds of its total depth, and allow the wax to begin to set for approximately 10 minutes. (C) Use an egg of the species that you will be injecting to make an impression of the egg in the paraffin wax and remove the egg after 1 minute. (D) Allow the paraffin wax to fully harden for at least 30 minutes.

Supplemental Figures

Supplemental video S1: Protocol for the rapid intravenous *in-ovo* injection of developing amniote embryos. Video demonstrating all steps required for the successful execution of this protocol.
